# Assessing knowledge and skills of maternity care professionals regarding neonatal hyperbilirubinaemia: a nationwide survey

**DOI:** 10.1186/s12884-020-03463-0

**Published:** 2021-01-19

**Authors:** Berthe A. M. van der Geest, Imke M. Theeuwen, Irwin K. M. Reiss, Eric A. P. Steegers, Jasper V. Been

**Affiliations:** 1 Department of Paediatrics, Division of Neonatology, Erasmus MC – Sophia Children’s Hospital, University Medical Centre Rotterdam, PO Box 2060, 3000 CB Rotterdam, Netherlands; 2 Department of Obstetrics and Gynaecology, Division of Obstetrics and Foetal Medicine, Erasmus MC, University Medical Centre Rotterdam, Rotterdam, the Netherlands; 3 Department of Public Health, Erasmus MC, University Medical Centre Rotterdam, Rotterdam, the Netherlands

**Keywords:** Neonatal hyperbilirubinaemia, Neonatal jaundice, Maternity care professionals, Maternity care assistants, Knowledge, Recognition, Experience, Expertise, Skills

## Abstract

**Background:**

Neonatal hyperbilirubinaemia is a physiologic phenomenon, but, when severe, may cause lifelong disability. Maternity care assistants (MCAs) play an important role in timely recognition of severe neonatal jaundice. We assessed knowledge and skills of MCAs regarding neonatal hyperbilirubinaemia.

**Methods:**

All Dutch MCAs (*n* = 9065) were invited to fill out a questionnaire assessing knowledge, expertise, and handling of neonatal jaundice. Additionally, we developed an e-learning and provided training sessions to a subgroup of MCAs (*n* = 99), and assessed their knowledge on neonatal hyperbilirubinaemia before and after the training.

**Results:**

One thousand four hundred sixty-five unique online questionnaires were completed (response 16.2%). The median number of correctly answered knowledge questions was 5 (out of six; IQR 1). Knowledge was significantly better when respondents had had in-service training on neonatal hyperbilirubinaemia in the previous year (*p* = 0.024). Although 82% of respondents felt highly skilled or skilled to assess jaundice, accuracy of estimation of total serum bilirubin levels by assessing skin colour was generally poor and prone to underestimation. Among participants attending a training session, those who completed the e-learning beforehand had higher pre-training scores (5 (IQR 1) vs. 4 (IQR 2); *p* < 0.001). The median post-training score was higher than pre-training (6 (IQR 1) vs. 5 (IQR 2); *p* < 0.001).

**Conclusions:**

Background knowledge of MCAs regarding neonatal hyperbilirubinaemia was adequate, but can be improved by further training. Estimation of total serum bilirubin levels based on skin colour was often inadequate. Approaches to improve timely recognition of jaundiced neonates are needed.

**Supplementary Information:**

The online version contains supplementary material available at 10.1186/s12884-020-03463-0.

## Background

Neonatal jaundice, caused by elevated total serum bilirubin (TSB) levels, occurs in approximately 60–80% of all live-born infants [1]. In Europe, 3.7 per 10,000 term and near term neonates develop severe hyperbilirubinaemia [2]. When TSB is highly elevated, bilirubin can cross the blood-brain barrier and may cause Kernicterus Spectrum Disorder (KSD) [3]. Initially, KSD presents as acute bilirubin encephalopathy, which in severe cases may progress to “classical kernicterus”. Although classical kernicterus is rare, consequences are severe and include permanent motor dysfunction, visual and hearing impairment, seizures, and sometimes mental retardation [4–6].

KSD and its devastating consequences are entirely preventable by timely recognition and treatment of potentially severe hyperbilirubinaemia. Some countries advise universal screening for neonatal hyperbilirubinaemia by quantifying TSB or transcutaneous bilirubin (TcB) levels at least once during the first week of life [7]. In most countries, including in the Netherlands, visual inspection by maternity care professionals is relied on as a first-line approach to identifying neonates requiring total bilirubin quantification [8, 9]. However, visual estimation of jaundice is known to be inaccurate and therefore ineffective in preventing KSD [8, 10, 11].

In the Netherlands, a significant proportion of neonates are born in a primary care setting or are discharged from the hospital within a few hours after birth [12]. A maternity care assistant (MCA, i.e. a skilled nurse with a lower secondary education degree) provides maternity care for the first 8 days under supervision of a community midwife [13]. If potentially severe hyperbilirubinaemia is suspected, the MCA will consult the community midwife, who is responsible for the mother and the neonate. The community midwife may then decide to draw blood to have TSB quantified (usually in a laboratory of a nearby hospital). If the TSB level indicates the need for treatment (usually phototherapy) or in case of another potential clinical problem [9], a paediatrician from a nearby hospital will be consulted. Traditionally, phototherapy is performed in the hospital.

Whereas theoretically entirely preventable, KSD still occurs in the Netherlands [14]. As MCAs have a first-line role in the recognition of potentially severe neonatal jaundice, we aimed to examine their current state of knowledge and skills regarding hyperbilirubinaemia. Accordingly, we propose recommendations for further training of maternity care professionals on the topic.

## Methods

### Study design

We conducted a nationwide online survey among MCAs in the Netherlands to assess their knowledge regarding neonatal hyperbilirubinaemia and their skills regarding its assessment. In addition, from MCAs working in primary care and participating in training sessions provided as part of the Screening and TreAtment to Reduce Severe Hyperbilirubinaemia in Infants in Primary care (STARSHIP) Trial (NTR7187), we collected paper-based questionnaires assessing knowledge on hyperbilirubinaemia and on STARSHIP study procedures [15]. The STARSHIP Trial is an ongoing factorial stepped-wedge cluster randomised controlled trial aimed at assessing the effectiveness of: 1. daily TcB screening to reduce the incidence of severe hyperbilirubinaemia, and 2. phototherapy provided in primary care birth centres to reduce the number of neonates admitted to hospital for hyperbilirubinaemia treatment [15, 16].

### Setting

#### Nationwide online survey

The online survey was designed to assess the current state of knowledge on pathophysiology, risk factors, recognition and treatment of neonatal hyperbilirubinaemia, and professional experience with neonatal hyperbilirubinaemia of MCAs across the Netherlands. The nationwide survey was distributed via the Knowledge Centre of Maternity Care (*Kenniscentrum Kraamzorg*; KCKZ; www.kckz.nl) via a weblink in their bimonthly newsletter, on the 29th of January 2018. In addition, the weblink to the survey was posted on KCKZ’s social media pages twice, on the 5th and the 13th of February 2018. The questionnaire was closed on the 28th of February 2018. The survey was constructed using LimeSurvey (version 2.06lts) [17].

#### STARSHIP training session questionnaire

Training sessions on neonatal hyperbilirubinaemia were provided to MCAs as part of the STARSHIP Trial [15]. Training sessions were aimed at increasing knowledge on pathophysiology, risk factors, recognition and treatment of neonatal hyperbilirubinaemia, and at providing information on STARSHIP study procedures (e.g. inclusion process, data collection). The training sessions were provided locally in each of the participating PCBCs.

Several weeks before and in preparation for each training session, all MCAs of the PCBCs were invited to complete an e-learning covering the same topics as the training session.

Immediately prior to and following each training session, a paper-based questionnaire was filled out by participating MCAs to assess their knowledge on hyperbilirubinemia and to assess changes in knowledge following the training session itself. Time to fill out the questionnaire was not limited.

### Participants

#### Online survey

The online survey was sent to all MCAs registered at KCKZ. At the time, 9065 MCAs were registered at KCKZ and all were subscribed to the electronic newsletter. These MCAs constitute 99.9% of all MCAs in the Netherlands. Approximately 99% of the MCAs registered at KCKZ work in primary care.

#### Training session questionnaire

The training session questionnaire was provided to all employees of the seven participating PCBCs who attended the training sessions of the STARSHIP Trial. The knowledge questions in the training session questionnaires were also used in the online survey. In order to prevent interference of the online questionnaire with the training session questionnaire, here we only report data from the training session questionnaires that had been filled out before the 29th of January 2018, i.e. the day the national questionnaire was published online.

### Data collection

#### Online survey

The online survey started with questions on baseline characteristics of the MCA: i.e. age (in years), educational level (six categories, based on the definition of Statistics Netherlands [18]), type of maternity care education, experience in perinatal care (in years), working area (province and place; this was converted to urban vs. non-urban; a non-urban working area was defined as category 5 (less than 500 addresses per square kilometer) of the classification of urbanity of Statistics Netherlands [19]), working location (at home; in a PCBC; in a hospital). See Additional files 1 and 2 for the Dutch and translated version of the online survey respectively.

Following the baseline characteristics, the online survey continued with questions to assess MCAs’ knowledge on pathophysiology, recognition and treatment of hyperbilirubinaemia, and questions assessing their professional experience with neonatal hyperbilirubinaemia. In addition, the questionnaire included three clinical case descriptions (with photos) of neonates with various degrees of jaundice. The photos displayed the neonate wearing a nappy only. Of each case two photos were shown: one of the neonate’s face and upper body (taken whilst gently stretching part of the neonate’s skin using two fingers to allow for better colour assessment) and one of the neonate’s whole body. All pictures were taken by an experienced professional medical photographer. Information on sex, postnatal age, gestational age, and risk factors for severe hyperbilirubinaemia were also given (case characteristics are displayed in Table 1). For each case, MCAs were asked to: 1. visually assess skin colour (pink, slightly yellow, moderately yellow, quite yellow, very yellow), 2. estimate TSB level (by choosing a TSB range: < 50 μmol/L (< 2.92 mg/dL), 50–100 μmol/L (2.92–5.85 mg/dL), 100–200 μmol/L (5.85–11.70 mg/dL), 200–300 μmol/L (11.70–17.54 mg/dL), 300–450 μmol/L (17.54–26.32 mg/dL), > 450 μmol/L (> 26.32 mg/dL), and 3. determine their action plan accordingly (no action; no action but active surveillance; consultation of midwife; consultation of midwife to collect blood sample for quantifying TSB level; emergency consultation of midwife to collect blood sample for quantifying TSB level; other (please specify)). Treatment guidelines for each case are determined by age, gestational age and risk status according to Dutch national multidisciplinary guideline [9].

**Table 1:** Characteristics of photo cases in online survey

Case	Sex	Ethnicity	Postnatal age (days)	Gestational age at birth (weeks)	Risk Factors^a^	TSB (μmol/L)	Threshold for phototherapy treatment (μmol/L) [20]
**1**	Male	Caucasian	3 (49–72 h)	38	None	256	270
**2**	Female	Non-Caucasian	3 (49–72 h)	41	None	145	290
**3**	Male	Caucasian	3 (49–72 h)	40	None	182	280

*TSB* total serum bilirubin

^a^Risk factors used in Dutch bilirubin nomogram: blood group antagonism; other haemolytic disease; asphyxia (Apgar score at 5 min < 5 or umbilical cord pH in pH < 7.0); ill, drowsy, or (suspicion of) infection or sepsis; serum albumin level < 30 g/L (if quantified) [20].

#### Training session questionnaire

The training questionnaire included baseline characteristics of the MCA (i.e. age (in years), experience in perinatal care (in years), educational level, type of maternity care education, working location (at home, in a primary care birth centre, in a hospital), whether or not the MCA was involved in doing intake interviews in the last year, and whether or not the MCA had assisted in deliveries in the last year), whether or not the MCA had (partially or fully) completed the e-learning, six questions on pathophysiology, recognition, and treatment of hyperbilirubinaemia, and five questions on the STARSHIP trial and its study procedures. In order to facilitate comparison of the results from the online survey and the training sessions, only the six multiple choice questions on pathophysiology, recognition, and treatment (i.e. the knowledge topics tested in the online survey as well) were analysed.

The questionnaire was used to assess a change in knowledge regarding neonatal hyperbilirubinaemia after the training session. For this purpose, two sets (A and B) were used. Within each set each question was matched to a similar, but not identical, question (i.e. questions on the same topic and of the same difficulty level) of the other question set. After the training session, the questionnaire with the other set of questions was filled out by the MCA to evaluate a change in knowledge following the training session. To account for potential differences in difficulty level of the questions between sets A and B, the two question sets were used alternately before and after the training session. That is, approximately half the participants completed set A before the session and set B after, while the other half first completed B and then A.

### Outcomes

#### Online survey

In case of duplicate responses to the online questionnaire, only the most recent response was included. Responses were defined as duplicate when either the submitted e-mail address or all baseline characteristics were exactly identical. In order to assess representativeness of the survey respondents, we compared the age and working area (province) distributions of the MCAs who filled out the online survey to the age distribution of all Dutch MCAs as provided by KCKZ, and to the province of residence of the Dutch population in general [21]. Level of knowledge on hyperbilirubinaemia was expressed as the number of questions answered correctly. Level of knowledge was defined as poor (≤2 correct answers out of six questions), moderate (3–4 correct answers), or good (5–6 correct answers).

For the three jaundice cases, descriptive statistics were used to describe visual assessment of the skin colour. For each case, we noted the number of ranges (see ‘Data collection’ above) by which MCAs were off when estimating TSB levels (i.e. correct estimation = off by zero ranges, estimation of one range higher or lower than correct TSB range = off by one range, etc.). The median number of ranges per MCA was calculated across the three cases.

The visual assessment of the skin colour was plotted against the MCA’s action plan and the estimated TSB level. For this purpose the three jaundice cases were taken together and the answers were treated like independent observations.

#### Training session questionnaire

Similar to the online survey, the level of knowledge was expressed as the number of correctly answered knowledge questions. For the training questionnaire, next to a wrong answer, answers were considered incorrect when: 1) the question was unanswered, 2) it was unclear or doubtful which answer was selected, or 3) more than one answer was selected.

Question sets A and B were used alternately. We compared the number of correctly answered questions between question sets A and B pre-training to identify whether there was a difference in difficulty between the sets.

To evaluate the change in knowledge regarding hyperbilirubinaemia following the training sessions, the difference in the number of correct answers before and after the training session was calculated.

### Statistical analysis

All data were analysed using SPSS Statistics (v25.0.0.1). Data are summarised using descriptive statistics. Mean and standard deviation (SD) are provided for continuous and (approximately) normally distributed data. Median and interquartile range (IQR) are provided for continuous, non-normally distributed data. Comparisons for non-normally distributed continuous data were performed using Wilcoxon signed rank test and Mann-Whitney U test. Categorical data were compared using χ2-tests or Fisher’s exact test as appropriate. Two-sided *p*-values of < 0.05 were considered to indicate statistical significance.

## Results

### Online survey

**Fig. 1:**
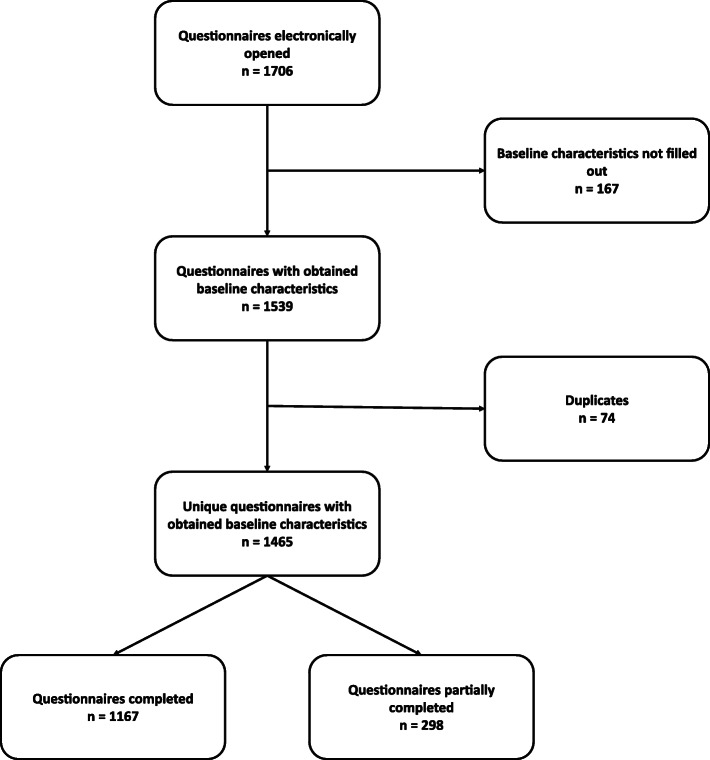
Flowchart online survey

The web-portal was accessed 1706 times. After removal of 74 duplicates, there were 1465 unique questionnaires with at least all baseline characteristics having been filled out. Data from 1167 completed and 298 partially completed questionnaires were analysed (in total 16.2% of all Dutch MCAs) (Fig. 1). Sample frequencies for age and province of working area of survey respondents were comparable to national data, indicating that our sample was representative (See Additional file 3).

There were no relevant differences in MCAs’ baseline characteristics according to whether an MCA had or had not fully completed the questionnaire. We used both completed questionnaires and completed sections of partially completed questionnaires for further analyses. Baseline characteristics are displayed in Table 2.

**Table 2:** Baseline characteristics of maternity care assistants attending the online survey by knowledge on hyperbilirubinaemia

	**Level of knowledge on hyperbilirubinaemia (*N*** **= 1366)**		
	Poor: ≤2 correct answers (*n* = 64) *mean (SD)*	Moderate: 3–4 correct answers (*n* = 515) *mean (SD)*	Good: 5–6 correct answers (*n* = 787) *mean (SD)*
**Age (years)**	50 (12)	49 (11)	47 (11)
**Experience (years)**	21 (16)	17 (12)	17 (11)
	***n(%)***	***n(%)***	***n(%)***
**Educational level**^a^			
**Low**	12 (19)	55 (11)	69 (9)
**Medium**	46 (72)	398 (78)	643 (82)
**High**	1 (2)	31 (6)	42 (5)
***Missing***	*5 (8)*	*31 (6)*	*33 (4)*
**Working at client’s home**			
**Yes**	63 (98)	505 (98)	741 (94)
**No**	1 (2)	10 (2)	46 (6)
**Working in a birth centre/hotel/clinic**			
**Yes**	2 (3)	33 (6)	75 (10)
**No**	62 (97)	482 (94)	712 (90)
**Working in a hospital**			
**Yes**	4 (6)	39 (2)	77 (10)
**No**	60 (94)	476 (98)	710 (90)
**Urbanisation level of working area**^**b**^			
**Urban**	53 (83)	470 (92)	734 (93)
**Rural**	3 (5)	17 (3)	12 (2)
**Unknown**	8 (13)	24 (5)	41 (5)
**Delivery assistant in the past 12 months**			
**Yes**	40 (63)	388 (75)	603 (77)
**No**	21 (33)	110 (21)	167 (21)
**Has been working for < 12 months**	3 (5)	17 (3)	17 (2)
**Had (in-service) training on hyperbilirubinaemia in the past 12 months**			
**Yes**	10 (19)	101 (21)	198 (26)
**No**	44 (81)	389 (79)	569 (74)
**Came in contact with neonate in need of treatment for hyperbilirubinaemia in the past 12 months**			
**Yes**	23 (42)	279 (57)	421 (55)
**No**	32 (58)	211 (43)	346 (45)

*MCA* maternity care assistant, *SD* standard deviation

^a^Educational level based on the definition of Statistics Netherlands [18]

^b^Rural working area is defined as category 5 of the classification of urbanisation of Statistics Netherlands [19]

#### Knowledge regarding hyperbilirubinaemia

One thousand three hundred sixty-six respondents completed all knowledge questions. The median number of correctly answered questions was 5 (out of 6; IQR 1). MCAs who had attended an in-service training on neonatal hyperbilirubinaemia in the last year (related or unrelated to the STARSHIP trial) were more likely to have a higher level of knowledge on hyperbilirubinaemia (i.e. low, medium, or high) than MCAs who had not attended an in-service training on neonatal hyperbilirubinaemia (*p* = 0.024; Table 2). The vast majority of MCAs (90%) felt that their knowledge on hyperbilirubinaemia was adequate. However, 63% of the MCAs still expressed an additional training need to increase their knowledge on hyperbilirubinaemia (Table 3).

**Table 3:** Frequencies of answers given to questions on experience with and current knowledge regarding neonatal hyperbilirubinaemia

	*N* = 1313 n (%)
**Number of times cared for a jaundiced neonate who had to be admitted to the hospital for hyperbilirubinaemia treatment in the last year**	
**Never**	589 (45)
**1–2 times**	575 (44)
**3–5 times**	104 (8)
**6–10 times**	20 (2)
**11–20 times**	11 (1)
**More than 20 times**	13 (1)
**Self assessed capability of recognising jaundice by MCA**	
**Totally incapable**	1 (0)
**Incapable**	9 (1)
**Neutral**	231 (18)
**Capable**	922 (70)
**Very capable**	150 (11)
**In my experience, jaundiced neonates are usually recognised and treated in time**	
**All neonates**	999 (76)
**Most of the neonates**	303 (23)
**Some neonates**	8 (1)
**No neonates**	2 (0)
***Missing***	*1 (0)*
**What are common causes for jaundice not being recognised in time** *(multiple options possible)*	
**Delay in recognition by MCA**	122 (39)
**Delay in recognition by midwife**	242 (77)
**Delay in TSB quantification**	44 (14)
**Delay due to consultation of paediatrician**	11 (4)
**Delay in transferring neonate to hospital**	28 (9)
**Other**	11 (4)
**My knowledge regarding neonatal hyperbilirubinaemia is:**	
**More than sufficient**	211 (16)
**Sufficient**	970 (74)
**Insufficient**	130 (10)
**Largely insufficient**	2 (0)
**I would like to learn more about neonatal hyperbilirubinaemia**	
**Yes**	820 (63)
**I do not know**	81 (6)
**No**	411 (31)

*MCA* maternity care assistant, *TSB* total serum bilirubin

#### Assessment of jaundice

Out of 1313 MCAs who completed the questionnaire’s section on their experience with jaundice assessment, 82% considered themselves very capable or capable to visually assess neonatal jaundice. Virtually all MCAs (99%) felt that the vast majority of neonates with hyperbilirubinaemia is diagnosed on time in the Netherlands. If a jaundiced neonate was not timely recognised, MCAs primarily identified insufficient visual assessment (i.e. late recognition or inaccurate visual assessment) by a midwife or MCA to be the main cause of late diagnosis. Other causes were identified less frequently: late/delayed blood sample collection (14%), late/delayed referral to secondary or tertiary care (9%), and late/delayed consultation with a paediatrician (4%). See Table 3.

**Fig. 2:**
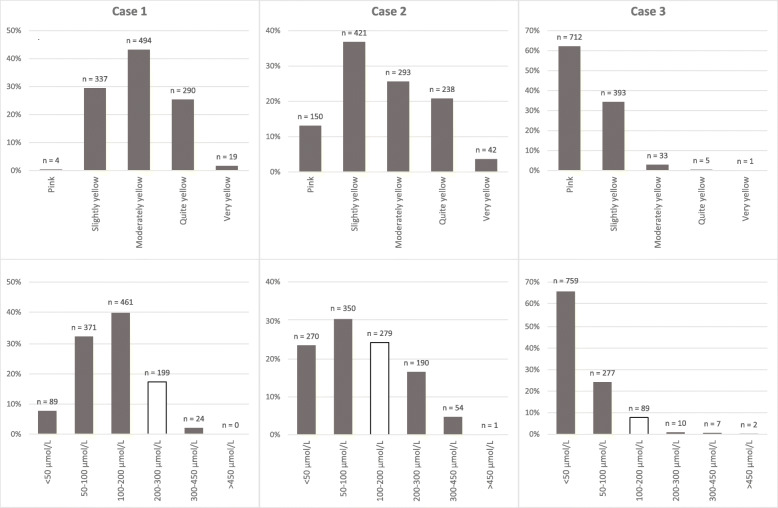
Visual skin colour assessment of photo cases (above) and estimated TSB level per photo case (below) by maternity care assistants who participated in the online survey. The white bars indicate the correct TSB range. TSB = total serum bilirubin; MCA = maternity care assistant

Results from visual assessment of the three cases of various degrees of jaundice by MCAs are shown in Fig. 2. There was substantial variation in the degree to which MCAs felt the cases did or did not have visible jaundice. Twelve MCAs noted that the skin colour made assessment more difficult in the non-Caucasian neonate.

Estimation of TSB range by MCAs based on visual inspection of the photographs was rarely in accordance with the actual TSB level in blood samples (Fig. 2). Only 22 of 1144 MCAs (2%) estimated TSB in the correct range for all three cases, and the majority (62%) did not estimate TSB range correctly in any of the cases. As shown in Fig. 2, there was a structural underestimation of TSB levels by MCAs across the three cases based on visual inspection. The adequacy of estimation of TSB was not different in MCAs having different levels of knowledge (*p* = 0.067).

**Fig. 3:**
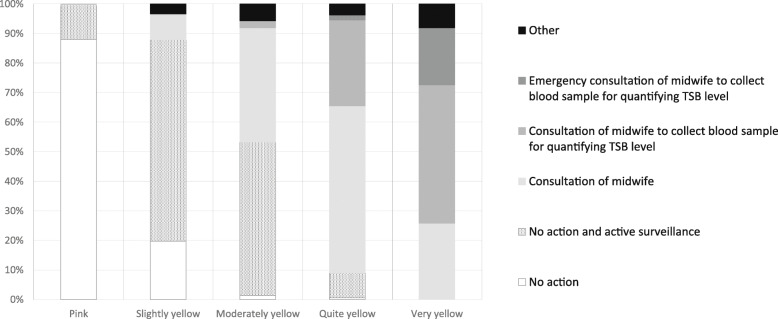
Maternity care assistant’s action plan according to neonate’s skin colour as assessed visually. TSB = total serum bilirubin

**Fig. 4:**
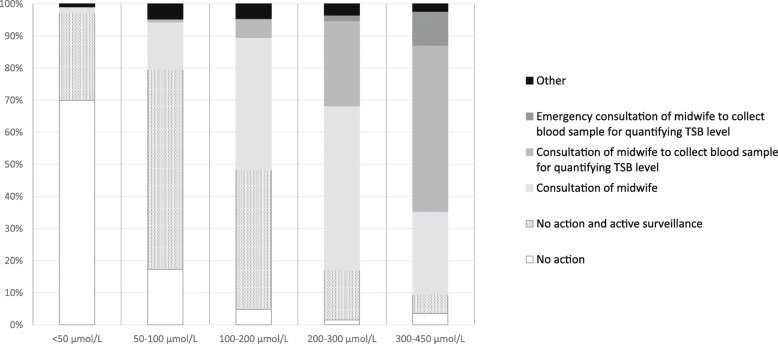
Maternity care assistant’s action plan according to estimated total serum bilirubin level. TSB = total serum bilirubin

**Fig. 5:**
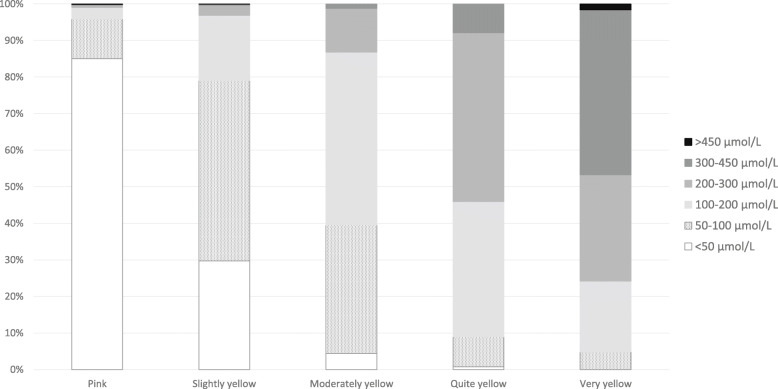
Estimated total serum bilirubin level according to estimated skin colour

When asked for their action plan, MCAs were more likely to choose ‘no action (and active surveillance)’ when they assessed the skin colour of the neonates in the three photo cases to be pink or only slightly yellow or if they estimated the TSB level to be low (*p* < 0.001; Figs. 3 and 4). The more yellow the MCAs assessed the skin colour of the neonates, the more likely they were to choose an active action plan. At the same time, a large proportion (63%) of the MCAs indicated that they would not have a blood sample collected for TSB quantification from a neonate despite their assessment indicating that the neonate was quite or very yellow. There was a positive association between MCAs’ assessment of the degree of jaundice and the estimated TSB range (Chi square for trend *p* < 0.001 for each case; Fig. 5).

**Fig. 6:**
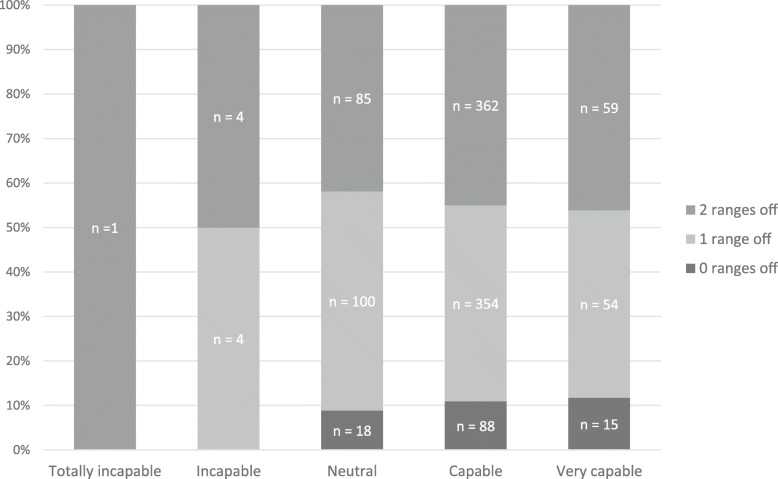
Self-assessed versus actual ability of maternity care assistants to estimate total serum bilirubin via visual inspection. Maternity care assistants (MCAs) were asked to estimate the total serum bilirubin (TSB) level of a neonate based on two photographs. For each case, we noted the number of 50 μl/L ranges by which MCAs were off when estimating TSB levels. The median number of ranges per MCA was calculated across the three cases. In this figure the self-assessed capability of recognising jaundiced neonates in time was plotted against the median number of ranges by which the specific MCA was off when estimating TSB level

There was no association between MCAs’ self-rated capability to assess jaundice and their actual ability to correctly estimate TSB ranges in the three cases based on visual inspection (Fig. 6; *p* = 0.794).

### Training session questionnaire

**Fig. 7:**
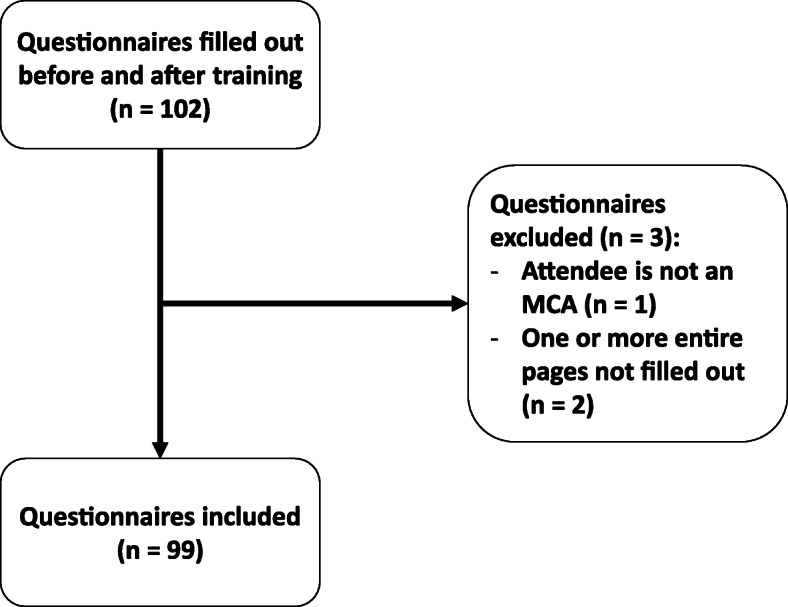
Flowchart of training session questionnaire inclusion. MCA = maternity care assistant

In December 2017 and January 2018 a total of 11 training sessions in five PCBCs were organised with a total of 102 attendees. Three questionnaires were excluded from further analysis as one attendee was not an MCA and two attendees did not fill out all pages of the questionnaire (Fig. 7).

Baseline characteristics of the respondents of the included questionnaires are displayed in Table 4.

**Table 4:** Baseline characteristics of maternity care assistants who filled out the training session questionnaire

	***N*** **= 99**
**Attendees per training session (n (%))**	11 (4)
**Age (years; mean (SD))**	44 (10)
**Experience as MCA (years; mean (SD))**	17 (10)
	%
**Work location**	
**Birth Hotel Haga**	24
**Birth Hotel Maasstad**	37
**Maternity Care Hotel Noord**	17
**Birth Centre Sophia**	7
**Birth Clinic Westeinde**	14
**Workplace(s)**	
**In a primary care birth centre/hotel/clinic**	92
**In a primary care birth centre/hotel/clinic and at client’s home**	8
**Delivery assistant**	
**Yes**	81
**No**	19
**Interviewer for client’s first intake**	
**Yes**	12
**No**	87
***Unknown***	1
**Completed e-learning before training session**	
**Yes**	71
**Partly**	12
**No**	16

*MCA* maternity care assistant

There was no relevant difference between the median pre-training scores for the two different question sets A and B (5 (IQR 1) vs. 5 (IQR 2), see Additional file 4), indicating that fair comparison of pre- and post-training scores was possible.

Median test scores were higher following the training session (5 (IQR 2) pre-training versus 6 (IQR 1) post-training; *p* < 0.001). There was no association between MCAs’ age or years of experience and improvement in the scores (*p* = 0.819 and *p* = 0.934). MCAs who had completed the e-learning prior to the actual training session had a higher median pre-training score than those who had not completed the e-learning (median score 5 (IQR 1) vs. 4 (IQR 2), respectively; *p* < 0.001). The post-training score did not differ between MCAs who had completed the e-learning and those who had not (median score 6 (IQR 1) vs. 6 (IQR 1), respectively; *p* = 0.656).

## Discussion

Being alert to identify term and near term neonates with potential hyperbilirubinaemia is daily practice for MCAs. In this study, we examined the knowledge and skills of MCAs regarding neonatal hyperbilirubinaemia. We found that background knowledge on neonatal hyperbilirubinaemia among MCAs was generally adequate, with further evidence indicating that this knowledge could be improved by training or e-learning. The estimation of TSB levels based on skin colour was, however, often inadequate, generally leading to underestimation.

There have been very few previous assessments of knowledge regarding neonatal hyperbilirubinaemia among maternity care professionals. We are aware of one Egyptean study, which demonstrated knowledge deficits among primary health care physicians in multiple areas, including screening methods, symptoms of complications, and treatment of hyperbilirubinaemia [22]. In the Netherlands, primary health care physicians are rarely involved in perinatal health care, limiting comparability of the findings of this study with ours.

Earlier studies have already demonstrated that visual inspection of jaundice is inaccurate for estimating the degree of hyperbilirubinaemia [7, 11]. Our results support this and also indicate that MCAs generally overestimated their own ability to estimate TSB via visual inspection. Their structural underestimation of TSB levels and their predominantly applied wait-and-see approach is particularly striking, whereby potentially severe neonatal hyperbilirubinaemia can be missed in these neonates. Missed diagnoses increase the risk of neonates going on to develop severe hyperbilirubinaemia and KSD, and there are multiple examples of this still happening in everyday perinatal care even in high-income countries including in the Netherlands [2, 23]. Hence, other approaches to timely identification of potentially severe hyperbilirubinaemia, such as TcB measurement, are needed. Our study also shows that MCAs whose level of knowledge on hyperbilirubinaemia was poor tended to have a lower ability to accurately estimate TSB levels based on visual inspection.

Together, this indicates that better knowledge on neonatal hyperbilirubinaemia may help increase MCAs’ awareness regarding the risks of potentially severe hyperbilirubinaemia and their accuracy of visual inspection. Knowledge can be increased further by training, as supported by various aspects of our study. In the national survey, knowledge was better when MCAs had received training on hyperbilirubinaemia in the previous year. Furthermore, pre-post training comparisons of MCAs’ knowledge on hyperbilirubinaemia in the STARSHIP training sessions indicate that these trainings, as well as the preceding e-learning, effectively increased MCAs’ knowledge. According to Lahti et al., e-learning can be as effective as traditional learning methods regarding knowledge, skills, and satisfaction of nurses and student nurses [24]. This suggests that e-learning can be used as an alternative for traditional training to further increase the knowledge of MCAs regarding neonatal hyperbilirubinaemia.

In the interpretation of our study it is important to consider a number of strengths and limitations. For the online survey, we were able to obtain a very large and – importantly − representative sample of all Dutch MCAs. Despite the large number of respondents, the response rate was only 16%. Other surveys among MCAs in the Netherlands have had response rates of 20–30% [25, 26]. The low response rate may have introduced nonresponse bias, as in that respondents might represent a selected sample of all MCAs in the Netherlands. Respondents may have been more interested in neonatal hyperbilirubinaemia beforehand than non-respondents, and may have had more knowledge on neonatal hyperbilirubinaemia, potentially leading to overestimation of the knowledge of MCAs regarding the topic. On the other hand, the realisation of MCAs that they lack knowledge on neonatal hyperbilirubinaemia and might learn something about it, may have resulted in overrepresentation of MCAs with less knowledge on neonatal hyperbilirubinaemia. It is not possible to assess the direction of any bias, if present, although the representativeness of the sample is somewhat reassuring. In the current study we were unable to collect data on knowledge and skills regarding neonatal hyperbilirubinaemia among all maternity care professionals in the Netherlands, such as obstetric nurses, midwives, obstetricians, and paediatricians. Nevertheless, as MCAs are mainly relied on for the first-line recognition of neonatal jaundice in the Dutch primary care setting, we consider these results highly relevant for clinical practice.

To allow assessment of knowledge and skills regarding neonatal hyperbilirubinaemia in a large cohort, we created a short online survey. This survey consisted of six knowledge questions covering different topics in the field of neonatal hyperbilirubinaemia. We acknowledge that the discriminative value of this short questionnaire to assess differences between the levels of knowledge among MCAs maybe somewhat limited, although the findings indicate that relevant differences in knowledge could in fact be identified and that these also appear to translate into variation in skills. In the online survey, we emphasised that the participants should not look up the correct answers, however we were unable to ensure that looking up the answers did not happen. If it did, this may have resulted in an overestimation of MCAs’ knowledge level and this should be taken into account when interpreting our results. Also it is important to note that our study may in itself have served to raise awareness of the importance of appropriate jaundice assessment and improved knowledge on the topic among MCAs through the Hawthorne effect [27].

Not only was the theoretical knowledge established in the online survey, but also clinical performance was tested using three cases of jaundice with photographs taken by a medical photographer. The colouration of these photographs may have differed among respondents according to the quality and the settings of their computer screen, although this is unlikely to have influenced our findings.

To the best of our knowledge, this is the first study to have investigated the knowledge and skills of MCAs regarding neonatal hyperbilirubinaemia. The findings indicate that MCAs overestimate their ability to assess TSB via visual inspection, at the same time confirming that visual inspection to assess hyperbilirubinaemia is inaccurate and prone to underestimation. We furthermore show that knowledge on hyperbilirubinaemia can be improved via training, potentially leading to improved ability to assess neonatal jaundice and to initiate appropriate action. Further research is needed to assess the knowledge and skills regarding neonatal hyperbilirubinaemia among other maternity care professionals, and to explore opportunities to improve recognition of neonatal hyperbilirubinaemia in the primary care setting, for example via screening programmes. Based on our findings, setting up regular training programmes for MCAs to update their knowledge and skills regarding neonatal hyperbilirubinaemia is recommended. Increased awareness among maternity care professionals caring for otherwise healthy neonates in primary care and more accurate approaches to recognition of hyperbilirubinaemia in these neonates are needed to help improve early recognition of potentially severe hyperbilirubinaemia and prevent the occurrence of KSD.

## Conclusions

In this national survey, knowledge of MCAs regarding neonatal hyperbilirubinaemia was generally adequate. Our findings indicate that knowledge can be improved further by an e-learning or providing training sessions. Visual assessment of jaundice was often inaccurate and structurally underestimated TSB levels. This confirms that, based on visual assessment, potentially severe neonatal hyperbilirubinaemia may be missed. This study suggests that increased awareness of the potential pitfalls of visual assessment and more accurate approaches are needed to improve early identification of potentially severe hyperbilirubinaemia.

## Supplementary Information

**Additional file 1.** Dutch version of online survey.

**Additional file 2.** Translated version of online survey (from Dutch to English).

**Additional file 3: Supplementary table 1.** Frequencies of age and province for maternity care assistants in the national survey sample and in the Netherlands.

**Additional file 4: Supplementary table 2.** Comparison of question sets of training session questionnaire.

## Data Availability

The datasets from the current study are available from the corresponding author on reasonable request.

## Abbreviations

IQR: Interquartile range
KCKZ: Kenniscentrum Kraamzorg (Knowledge Centre of Maternity Care)
KSD: Kernicterus spectrum disorder
MCA: Maternity care assistant
SD: Standard deviation
STARSHIP Trial: Screening and TreAtment to Reduce Severe Hyperbilirubinaemia in Infants in Primary care Trial
TcB: Transcutaneous bilirubin
TSB: Total serum bilirubin
