# Supplementary material for: Assessing knowledge and skills of maternity care professionals regarding neonatal hyperbilirubinaemia: a nationwide survey
Source: BMC Pregnancy Childbirth. 2021 Jan 19;21:63. doi: 10.1186/s12884-020-03463-0 (PMC7814718; doi:10.1186/s12884-020-03463-0)
Supplement: Supplementary file 1 — Additional file 1. Dutch version of online survey. [file 12884_2020_3463_MOESM1_ESM.docx]

**Additional file 1: Dutch version of online survey**

**Geelzucht bij pasgeborenen; kennis van kraamverzorgenden**

Algemeen

1. Bent u op dit moment werkzaam als kraamverzorgende?
   1. Ja
   2. Nee
2. Werkt u in Nederland?
   1. Ja
   2. Nee

Uw gegevens

1. Wat is uw leeftijd? ____ jaar
2. Wat is het niveau van uw hoogst afgeronde opleiding?
   1. VMBO
   2. HAVO
   3. VWO
   4. MBO
   5. HBO
   6. WO
   7. Anders, namelijk
3. Welke opleiding tot kraamverzorgende heeft u gevolgd?
4. Verzorgende IG met uitstroomvariant/branche verbijzondering van Kraamzorg
5. Verzorgende IG zonder kraamdifferentiatie met aanvullende opleiding 311/313 en 650 uur BPV (beroepspraktijkvorming)
6. Brancheopleiding kraamverzorgende
7. MDGO-VZ, uitstroom kraam
8. Verkorte opleiding kraamverzorgende met erkend certificaat ROC, verplichte BPV 650 uur
9. Internaat (kraamverzorgende oude stijl tot 1987)
10. OVDB
11. Rijkskweekschool voor vroedvrouwen
12. Ik ben nog in opleiding
13. Anders, namelijk:
14. Hoeveel jaar bent u werkzaam als kraamverzorgende? ___ jaar
15. In welke provincie werkt u (voornamelijk)?
16. Drenthe
17. Flevoland
18. Friesland
19. Gelderland
20. Groningen
21. Limburg
22. Noord-Brabant
23. Noord-Holland
24. Overijssel
25. Utrecht
26. Zeeland
27. Zuid-Holland
28. Wat is de plaatsnaam van uw voornaamste werklocatie?
29. Waar werkt u als kraamverzorgende? *Meerdere antwoorden mogelijk*
30. Bij mensen thuis
31. In een geboortecentrum/geboortehotel/geboortekliniek/kraamhotel
32. In het ziekenhuis
33. Heeft u in het afgelopen jaar partusassistentie als kraamverzorgende verricht?
34. Ja
35. Nee
36. Ik ben korter dan een jaar werkzaam als kraamverzorgende, maar ik heb wel partusassistentie gedaan.
37. Ik ben korter dan een jaar werkzaam als kraamverzorgende en ik heb geen partusassistentie gedaan.
38. Heeft u in het afgelopen jaar intakegesprekken gedaan voor de kraamzorg?
39. Ja
40. Nee
41. Ik ben korter dan een jaar werkzaam als kraamverzorgende, maar ik heb wel intakegesprekken gedaan.
42. Ik ben korter dan een jaar werkzaam als kraamverzorgende en ik heb geen intakegesprekken gedaan.
43. Deze vragenlijst hangt samen met een onderzoek over hyperbilirubinemie bij pasgeborenen dat in een aantal centra in Nederland wordt opgezet. Werkt u in een van de deelnemende centra van de STARSHIP Trial?
44. Ja *(ga naar vraag 13)*
45. Nee *(ga naar vraag 16)*

Onderzoek hyperbilirubinemie

1. In welk centrum werkt u?
   1. GeboorteHotel Maasstad, Rotterdam
   2. Kraamzorghotel Noord, Rotterdam
   3. Geboortecentrum Sophia, Rotterdam
   4. GeboorteHotel Haga, Den Haag
   5. Geboortekliniek Westeinde, Den Haag
   6. Geboortecentrum Livive, Tilburg
   7. Kraamafdeling Isala Kliniek, Zwolle
2. Heeft u de e-learning gemaakt over het onderzoek naar hyperbilirubinemie bij pasgeborenen in de eerste lijn?
   1. Ja, volledig
   2. Ja, deels
   3. Nee, maar de e-learning is wel naar mij verstuurd
   4. Nee, ik heb (nog) geen e-learning ontvangen
3. Heeft u de training op locatie gevolgd over het onderzoek naar hyperbilirubinemie bij pasgeborenen in de eerste lijn?
   1. Ja, volledig
   2. Ja, deels
   3. Nee

Kennis hyperbilirubinemie

1. Waar komt bilirubine in het bloed vandaan?
   1. Bilirubine ontstaat bij een tekort aan rode bloedcellen.
   2. Bilirubine ontstaat bij een tekort aan witte bloedcellen.
   3. Bilirubine ontstaat bij de afbraak van rode bloedcellen.
   4. Bilirubine ontstaat bij de afbraak van witte bloedcellen.
2. Wat is geen mogelijke oorzaak van hyperbilirubinemie?
   1. Een bloeduitstorting bij de baby, ontstaan bij de geboorte
   2. Bloedgroepantagonisme (moeder en baby hebben niet dezelfde bloedgroep)
   3. Het eten van veel wortelen tijdens de zwangerschap
   4. Een verstopte darm bij het kind
3. Hoe vaak komt het voor dat een baby geel ziet?
   1. Vaak; meer dan de helft van de baby's wordt geel
   2. Regelmatig, iets minder dan de helft van de baby's wordt geel
   3. Zelden, baby's worden bijna nooit geel
4. Wat is geen alarmsymptoom van hyperbilirubinemie?
   1. Geelzucht binnen 24 uur na de geboorte
   2. Veel drinken
   3. Suf zijn
   4. Liggen met een holle rug
5. Welke blijvende schade kan ontstaan door hyperbilirubinemie?
   1. Kaalheid
   2. Gele kleuring van de huid
   3. Onvruchtbaarheid
   4. Doofheid
6. Welke behandeling kan een arts gebruiken bij hyperbilirubinemie?
   1. Fototherapie, ook wel lichttherapie
   2. Alleen bijvoeden
   3. Baby bij het raam leggen
   4. Alle bovenstaande antwoorden zijn goed.

Uw ervaring met hyperbilirubinemie

1. Hoe vaak heeft u de afgelopen 12 maanden een baby met geelzucht gezien, die daarvoor behandeling nodig heeft gehad in het ziekenhuis?
   1. Nooit
   2. 1 of 2 keer
   3. 3 tot 5 keer
   4. 6 tot 10 keer
   5. 11 tot 20 keer
   6. Meer dan 20 keer
2. Heeft u in de afgelopen 12 maanden (bij)scholing gehad over geelzucht bij baby's?
   1. Ja, alleen voor het onderzoek naar de huidmeter en fototherapie op mijn werklocatie (zie helptekst hieronder)
   2. Ja, maar niet voor het hierboven genoemde onderzoek
   3. Ja, zowel de scholing voor het hierboven genoemde onderzoek als een andere (bij)scholing/andere (bij)scholingen
   4. Nee
3. Als kraamverzorgende beoordeelt u de kleur van de huid en kijkt u of het kind geel ziet. Hoe deskundig voelt u zich om (de mate van) geelzucht vast te stellen?
   1. Zeer deskundig
   2. Deskundig
   3. Neutraal
   4. Niet deskundig
   5. Helemaal niet deskundig
4. Naar uw mening, welk deel van de baby’s met ernstige geelzucht waar u voor zorgt, wordt op tijd gehandeld?
   1. Bij alle baby's waar ik voor zorg, wordt op tijd gehandeld. *(ga naar vraag 27)*
   2. Bij de meeste baby's waar ik voor zorg, wordt op tijd gehandeld. *(ga naar vraag 26)*
   3. Bij weinig baby's waar ik voor zorg, wordt op tijd gehandeld. *(ga naar vraag 26)*
   4. Bij geen enkele baby waar ik voor zorg, wordt op tijd gehandeld. *(ga naar vraag 26)*
5. Als er niet op tijd wordt gehandeld bij ernstige geelzucht, aan welke factor ligt dit dan voornamelijk? *1 of 2 antwoorden mogelijk.*
   1. Moeilijke of verkeerde inschatting van de ernst van de geelzucht door u of een andere kraamverzorgende
   2. Moeilijke of verkeerde inschatting van de ernst van de geelzucht door de verloskundige
   3. Vertraging door bloed prikken (moeilijk te prikken, lang wachten op iemand om te prikken, lang wachten op de uitslag, enz.)
   4. Vertraging door overleg met de kinderarts
   5. Moeite met/vertraging bij het doorsturen van de baby voor behandeling
   6. Anders, namelijk:
6. Naar uw mening, weet u voldoende over geelzucht en hyperbilirubinemie bij pasgeborenen?
   1. Ja, ruim voldoende
   2. Ja, voldoende
   3. Nee, onvoldoende
   4. Nee, ruim onvoldoende
7. Zou u meer willen weten over geelzucht en hyperbilirubinemie bij pasgeborenen?
   1. Ja
   2. Weet ik niet
   3. Nee
8. Wat zou u nog willen weten/willen leren over geelzucht en hyperbilirubinemie bij pasgeborenen?
9. Wanneer u met de verloskundige overlegt of er bloed geprikt moet worden bij een gele baby, hoe vaak zit u dan met de verloskundige op één lijn?
   1. Altijd *(ga naar vraag 32)*
   2. Regelmatig *(ga naar vraag 31)*
   3. Af en toe *(ga naar vraag 31)*
   4. Zelden *(ga naar vraag 31)*
   5. Nooit *(ga naar vraag 31)*
   6. Ik overleg niet met de verloskundige over een baby die geel ziet.  *(ga naar vraag 32)*
10. U heeft aangegeven niet altijd met de verloskundige op één lijn te zitten. Waar ligt dit meestal aan in uw ogen?
    1. De verloskundige onderneemt over het algemeen sneller actie bij een baby met geelzucht dan ik zou doen.
    2. De verloskundige onderneemt over het algemeen minder vaak actie bij een baby met geelzucht dan ik zou doen.
    3. Anders, namelijk:
11. Heeft u nog opmerkingen over de samenwerking met de verloskundige met betrekking tot geelzucht bij pasgeborenen?

Foto’s van pasgeborenen

*Pasgeborene 1*

Hieronder ziet u twee afbeeldingen van dezelfde baby. Het is een jongen van XX uur oud (tussen de 49 en 72 uur), geboren na een zwangerschap van 38+X weken. De bevalling is ingeleid. Verder zijn er geen bijzonderheden. Er zijn geen risicofactoren voor geelzucht bij dit kind.

Foto 1 van pasgeborene 1: foto van het gehele lichaam

*Er is geen toestemming voor het verspreiden van identificerende gegevens en foto’s van de baby’s voor andere doeleinden dan het tonen van de foto’s in de vragenlijst.*

Foto 2 van pasgeborene 1: foto van het gezicht en bovenlichaam van de baby (genomen terwijl een deel van de huid van de baby zachtjes gerekt wordt om een betere inschatting van de kleur mogelijk te maken)

*Er is geen toestemming voor het verspreiden van identificerende gegevens en foto’s van de baby’s voor andere doeleinden dan het tonen van de foto’s in de vragenlijst.*

1. Wat vindt u van de kleur van de huid?
   Ik beoordeel dit als…
2. Roze
3. Vleugje geel
4. Matig geel
5. Redelijk geel
6. Zeer geel
7. Op basis van de kleur van de huid, wat zou u doen met deze baby als u voor deze baby zou zorgen?
   1. Ik zou geen actie ondernemen.
   2. Ik zou nu geen actie ondernemen, maar de kleur van de huid zeer goed in de gaten houden.
   3. Ik zou de verloskundige om advies vragen.
   4. Ik zou met de verloskundige overleggen om bloed te prikken.
   5. Ik zou zo snel mogelijk de verloskundige bellen om met spoed bloed te prikken.
   6. Anders, namelijk:
8. Stel dat er bij deze baby bloed wordt geprikt, welke waarde zou u dan verwachten van het bilirubine-gehalte in het bloed?
   1. Minder dan 50 μmol/L
   2. Tussen de 50 en 100 μmol/L
   3. Tussen de 100 en 200 μmol/L
   4. Tussen de 200 en 300 μmol/L
   5. Tussen de 300 en 450 μmol/L
   6. Meer dan 450 μmol/L

*Pasgeborene 2*

Hieronder ziet u twee afbeeldingen van dezelfde baby. Het is een meisje van XX uur oud (tussen de 49 en 72 uur), geboren na een zwangerschap van 41+X weken. Er zijn geen bijzonderheden. Er zijn geen risicofactoren voor geelzucht bij dit kind.

Foto 1 van pasgeborene 2: foto van het gehele lichaam

*Er is geen toestemming voor het verspreiden van identificerende gegevens en foto’s van de baby’s voor andere doeleinden dan het tonen van de foto’s in de vragenlijst.*

Foto 2 van pasgeborene 2: foto van het gezicht en bovenlichaam van de baby (genomen terwijl een deel van de huid van de baby zachtjes gerekt wordt om een betere inschatting van de kleur mogelijk te maken)

*Er is geen toestemming voor het verspreiden van identificerende gegevens en foto’s van de baby’s voor andere doeleinden dan het tonen van de foto’s in de vragenlijst.*

1. Wat vindt u van de kleur van de huid?
   Ik beoordeel dit als…
2. Roze
3. Vleugje geel
4. Matig geel
5. Redelijk geel
6. Zeer geel
7. Op basis van de kleur van de huid, wat zou u doen met deze baby als u voor deze baby zou zorgen?
   1. Ik zou geen actie ondernemen.
   2. Ik zou nu geen actie ondernemen, maar de kleur van de huid zeer goed in de gaten houden.
   3. Ik zou de verloskundige om advies vragen.
   4. Ik zou met de verloskundige overleggen om bloed te prikken.
   5. Ik zou zo snel mogelijk de verloskundige bellen om met spoed bloed te prikken.
   6. Anders, namelijk:
8. Stel dat er bij deze baby bloed wordt geprikt, welke waarde zou u dan verwachten van het bilirubine-gehalte in het bloed?
   1. Minder dan 50 μmol/L
   2. Tussen de 50 en 100 μmol/L
   3. Tussen de 100 en 200 μmol/L
   4. Tussen de 200 en 300 μmol/L
   5. Tussen de 300 en 450 μmol/L
   6. Meer dan 450 μmol/L

*Pasgeborene 3*

Hieronder ziet u twee afbeeldingen van dezelfde baby. Het is een jongen van XX uur oud (tussen de 49 en 72 uur), geboren na een zwangerschap van 40+X weken. Er zijn geen bijzonderheden. Er zijn geen risicofactoren voor geelzucht bij dit kind.

Foto 1 van pasgeborene 2: foto van het gehele lichaam

*Er is geen toestemming voor het verspreiden van identificerende gegevens en foto’s van de baby’s voor andere doeleinden dan het tonen van de foto’s in de vragenlijst.*

Foto 2 van pasgeborene 2: foto van het gezicht en bovenlichaam van de baby (genomen terwijl een deel van de huid van de baby zachtjes gerekt wordt om een betere inschatting van de kleur mogelijk te maken)

*Er is geen toestemming voor het verspreiden van identificerende gegevens en foto’s van de baby’s voor andere doeleinden dan het tonen van de foto’s in de vragenlijst.*

1. Wat vindt u van de kleur van de huid?
   Ik beoordeel dit als…
2. Roze
3. Vleugje geel
4. Matig geel
5. Redelijk geel
6. Zeer geel
7. Op basis van de kleur van de huid, wat zou u doen met deze baby als u voor deze baby zou zorgen?
   1. Ik zou geen actie ondernemen.
   2. Ik zou nu geen actie ondernemen, maar de kleur van de huid zeer goed in de gaten houden.
   3. Ik zou de verloskundige om advies vragen.
   4. Ik zou met de verloskundige overleggen om bloed te prikken.
   5. Ik zou zo snel mogelijk de verloskundige bellen om met spoed bloed te prikken.
   6. Anders, namelijk:
8. Stel dat er bij deze baby bloed wordt geprikt, welke waarde zou u dan verwachten van het bilirubine-gehalte in het bloed?
   1. Minder dan 50 μmol/L
   2. Tussen de 50 en 100 μmol/L
   3. Tussen de 100 en 200 μmol/L
   4. Tussen de 200 en 300 μmol/L
   5. Tussen de 300 en 450 μmol/L
   6. Meer dan 450 μmol/L
