# Supplementary material for: Assessing knowledge and skills of maternity care professionals regarding neonatal hyperbilirubinaemia: a nationwide survey
Source: BMC Pregnancy Childbirth. 2021 Jan 19;21:63. doi: 10.1186/s12884-020-03463-0 (PMC7814718; doi:10.1186/s12884-020-03463-0)
Supplement: Supplementary file 2 — Additional file 2. Translated version of online survey (from Dutch to English). [file 12884_2020_3463_MOESM2_ESM.docx]

**Additional file 2: Translated version of online survey**

**Jaundice in newborns; knowledge of maternity care assistants**

General

1. Do you currently work as a maternity care professional?
   1. Yes
   2. No
2. Do you work in the Netherlands
   1. Yes
   2. No

Your characteristics

1. What is your age? ____ years
2. What is the highest level of education you have completed?
   1. Pre- vocational secondary education
   2. Senior general secondary education
   3. Pre-university education
   4. Secondary vocational education
   5. Higher professional education
   6. University education
3. Which training for maternity care assistants have you done?
   1. Nurse assistant individual care with branch specification of maternity care
      “Verzorgende IG met uitstroomvariant/branche verbijzondering van Kraamzorg”
   2. Nurse assistant individual care without maternity care differentiation with additional training 311/313 and 650 hours practical training
      “Verzorgende IG zonder kraamdifferentiatie met aanvullende opleiding 311/313 en 650 uur BPV (beroepspraktijkvorming)”
   3. Maternity care assistant branch training
      “Brancheopleiding kraamverzorgende”
   4. Secondary vocational education, sector services and healthcare, health department, care, outflow maternity care
      “MDGO-VZ, uitstroom kraam”
   5. Shortened training for maternity care assistants with a recognised ROC certificate, mandatory practical training 650 hours
      “Verkorte opleiding kraamverzorgende met erkend certificaat ROC, verplichte BPV 650 uur”
   6. Boarding school (maternity care assistant old style, until 1987)
      “Internaat (kraamverzorgende oude stijl tot 1987)”
   7. Training for care and service professions
      “OVDB”
   8. State training college for midwives
      “Rijkskweekschool voor vroedvrouwen”
   9. I am currently in training
   10. Other, please specify:
4. How many years have you been working as a maternity care assistant? ___ years
5. In which province do you work (mainly)?
   1. Drenthe
   2. Flevoland
   3. Friesland
   4. Gelderland
   5. Groningen
   6. Limburg
   7. Noord-Brabant
   8. Noord-Holland
   9. Overijssel
   10. Utrecht
   11. Zeeland
   12. Zuid-Holland
6. In which city/town do you work (mainly)?
7. Where do you work as maternity care assistant? *Multiple options possible*
   1. At client’s home
   2. In a primary care birth centre
   3. In a hospital
8. Did you provide delivery assistance in the past year?
   1. Yes
   2. No
   3. I have been working as maternity care assistant for less than one year, but I have provided delivery assistance
   4. I have been working as maternity care assistant for less than one year and I have not provided delivery assistance
9. Did you conduct maternity care intake interviews in the past year?
   1. Yes
   2. No
   3. I have been working as maternity care assistant for less than one year, but I have conducted intake interviews
   4. I have been working as maternity care assistant for less than one year and I have not conducted intake interviews
10. Do you work in one of the participating primary care birth centres of the STARSHIP Trial?
    1. Yes *(go to question 13)*
    2. No *(go to question 16)*

Research hyperbilirubinaemia

1. In which primary care birth centre do you work?
   1. Primary care birth hotel Maasstad, Rotterdam
   2. Maternity care hotel Noord, Rotterdam
   3. Primary care birth centre Sophia, Rotterdam
   4. Primary care birth hotel Haga, Den Haag
   5. Primary care birth clinic Westeinde, Den Haag
   6. Primary care birth centre Livive, Tilburg
   7. Maternity ward Isala, Zwolle
2. Did you complete the e-learning regarding research on neonatal hyperbilirubinaemia in primary care?
   1. Yes, in its entirety
   2. Yes, partly
   3. No, but I did receive the web link of the e-learning
   4. No, I have not received the web link to the e-learning
3. Have you attended an in-service training regarding research on neonatal hyperbilirubinaemia in primary care?
   1. Yes, completely
   2. Yes, partly
   3. No

Knowledge hyperbilirubinaemia

1. Where does bilirubin come from?
   1. Bilirubin originates from a shortage in red blood cells
   2. Bilirubin originates from a shortage in white blood cells
   3. Bilirubin originates from the breakdown of red blood cells
   4. Bilirubin originates from the breakdown of white blood cells
2. Which of the following is not a potential cause of hyperbilirubinaemia?
   1. A bruise in the baby, caused during birth
   2. Blood group antagonism (mother and baby do not have the same blood group)
   3. Eating many carrots during pregnancy
   4. A bowel obstruction in the neonate
3. How common is it for a baby to be jaundiced?
   1. Very common; more than half of the babies becomes jaundiced
   2. Often; slightly less than half of the babies becomes jaundiced
   3. Rarely; babies almost never become jaundiced
4. Which of the following is not a red flag when regarding hyperbilirubinaemia?
   1. Jaundice occurring within 24 hours after birth
   2. Drinking a lot
   3. Being drowsy
   4. Lying with arched neck and trunk
5. What permanent damage can result from hyperbilirubinaemia?
   1. Baldness
   2. Jaundiced skin tone
   3. Infertility
   4. Deafness
6. Which treatment can a doctor apply for hyperbilirubinaemia?
   1. Phototherapy, also known as light therapy
   2. Supplemental feeding
   3. Putting the baby near the window
   4. All of the above

Your experience with hyperbilirubinaemia

1. How often have you seen a jaundiced neonate necessitating treatment in the hospital in the past 12 months?
   1. Never
   2. 1 to 2 times
   3. 3 to 5 times
   4. 6 to 10 times
   5. 11 to 20 times
   6. More than 20 times
2. Have you attended an in-service training about neonatal jaundice in the past 12 months?
   1. Yes, only related to the study about the skin device and phototherapy at my work location
   2. Yes, but not related to the aforementioned study
   3. Yes, both a training related to the aforementioned study and other training sessions
   4. No
3. How competent do you feel in assessing (the degree of) neonatal jaundice?
   1. Very competent
   2. Competent
   3. Neutral
   4. Not very competent
   5. Not at all competent
4. In your opinion, which proportion of newborns with severe jaundice you care for is recognised in time?
   1. All babies I care for are timely recognised and treated *(go to question 27)*
   2. Most babies I care for are timely recognised and treated *(go to question 26)*
   3. Few babies I care for are timely recognised and treated *(go to question 26)*
   4. No babies I care for are timely recognised and treated *(go to question 26)*
5. If action is not taken in time when newborns are severely jaundiced, what is the main cause? *1 or 2 answers possible*
   1. Difficult or inadequate assessment of severity of jaundice by you or another maternity care assistant
   2. Difficult or inadequate assessment of severity of jaundice by the midwife
   3. Delay in total serum bilirubin quantification (difficulties during heel prick, long waiting time for the person who’s performing the heel prick, long waiting time for the result)
   4. Delay in consultation of a paediatrician
   5. Difficulties with/delay in admission of the neonate to the hospital
   6. Other, please specify:
6. In your opinion, do you have sufficient knowledge about neonatal hyperbilirubinaemia?
   1. Yes, more than sufficient
   2. Yes, sufficient
   3. No, insufficient
   4. No, very insufficient
7. Would you like to learn more about neonatal jaundice and hyperbilirubinaemia?
   1. Yes *(go to question 29)*
   2. I do not know *(go to question 30)*
   3. No *(go to question 30)*
8. What would you like to learn about neonatal jaundice and hyperbilirubinaemia?
9. When you consult a midwife or total serum bilirubin needs to be quantified in a jaundiced neonate, how often do you and the midwife agree?
   1. Always *(go to question 32)*
   2. Usually *(go to question 31)*
   3. Occasionally *(go to question 31)*
   4. Very rarely *(go to question 31)*
   5. Never *(go to question 31)*
   6. I do not consult a midwife about a jaundiced baby *(go to question 32)*
10. You have indicated that you and the midwife do not always agree. What is most often the cause of this?
    1. The midwife tends to take action sooner than I would do when a neonate is jaundiced.
    2. The midwife tends to take action later than I would do when a neonate is jaundiced.
11. Do you have any remarks regarding your collaboration with the midwife in relation to neonatal jaundice?

Photographs of newborns

*Newborn 1*

Below, two photographs of the same baby are displayed. It is a boy, he is XX hours (between 49 and 72 hours) old and born at a gestational age of 38+X weeks. The delivery was induced. There are no further abnormalities. There are no risk factors for neonatal jaundice.

Photo 1 of newborn 1: photo of baby’s whole body

*No informed consent for distribution of identifying characteristics or photographs other than via the questionnaire was obtained.*

Photo 2 of newborn 1: photo of the baby’s face and upper body (taken whilst gently stretching part of the baby’s skin using two fingers to allow for better colour assessment)

*No informed consent for distribution of identifying characteristics or photographs other than via the questionnaire was obtained.*

1. How would you assess the skin colour?
   I would assess this as…
   1. Pink
   2. Slightly yellow
   3. Moderately yellow
   4. Quite yellow
   5. Very yellow
2. Based on the skin colour, what would you do if you would take care of this baby?
   1. I would not do anything
   2. I would not do anything at the moment, but I would watch the skin colour very closely
   3. I would consult the midwife
   4. I would consult the midwife for bilirubin quantification
   5. I would consult the midwife immediately for urgent bilirubin quantification
   6. Other, please specify:
3. If bilirubin was quantified in this baby, what level of bilirubin in the blood would you expect?
   1. Less than 50 µmol/L (<2.92 mg/dL)
   2. Between 50 and 100 µmol/L (2.92 – 5.85 mg/dL)
   3. Between 100 and 200 µmol/L (5.85 – 11.7 md/dL)
   4. Between 200 and 300 µmol/L (11.7 – 17.54 mg/dL)
   5. Between 300 and 450 µmol/L (17.54 – 26.32 mg/dL)
   6. More than 450 µmol/L (>26.32 mg/dL)

*Newborn 2*

Below, two photographs of the same baby are displayed. It is a girl, she is XX hours (between 49 and 72 hours) and born at a gestational age of 41+X weeks. There are no abnormalities. There are no risk factors for neonatal jaundice.

Photo 1 of newborn 2: photo of baby’s whole body

*No informed consent for distribution of identifying characteristics or photographs other than via the questionnaire was obtained.*

Photo 2 of newborn 2: photo of the baby’s face and upper body (taken whilst gently stretching part of the baby’s skin using two fingers to allow for better colour assessment)

*No informed consent for distribution of identifying characteristics or photographs other than via the questionnaire was obtained.*

1. How would you assess the skin colour?
   I would assess this as…
   1. Pink
   2. Slightly yellow
   3. Moderately yellow
   4. Quite yellow
   5. Very yellow
2. Based on the skin colour, what would you do if you would take care of this baby?
3. I would not do anything
4. I would not do anything at the moment, but I would watch the skin colour very closely
5. I would consult the midwife
6. I would consult the midwife for bilirubin quantification
7. I would consult the midwife immediately for urgent bilirubin quantification
8. Other, please specify:
9. If bilirubin was quantified in this baby, what level of bilirubin in the blood would you expect?
10. Less than 50 µmol/L (<2.92 mg/dL)
11. Between 50 and 100 µmol/L (2.92 – 5.85 mg/dL)
12. Between 100 and 200 µmol/L (5.85 – 11.7 md/dL)
13. Between 200 and 300 µmol/L (11.7 – 17.54 mg/dL)
14. Between 300 and 450 µmol/L (17.54 – 26.32 mg/dL)
15. More than 450 µmol/L (>26.32 mg/dL)

*Newborn 3*

Below, two photographs of the same baby are displayed. It is a boy, he is XX hours (between 49 and 72 hours) and born at a gestational age of 40+X weeks. There are no abnormalities. There are no risk factors for neonatal jaundice.

Photo 1 of newborn 3: photo of baby’s whole body

*No informed consent for distribution of identifying characteristics or photographs other than via the questionnaire was obtained.*

Photo 2 of newborn 3: photo of the baby’s face and upper body (taken whilst gently stretching part of the baby’s skin using two fingers to allow for better colour assessment)

*No informed consent for distribution of identifying characteristics or photographs other than via the questionnaire was obtained.*

1. How would you assess the skin colour?
   I would assess this as…
   1. Pink
   2. Slightly yellow
   3. Moderately yellow
   4. Quite yellow
   5. Very yellow
2. Based on the skin colour, what would you do if you would take care of this baby?
3. I would not do anything
4. I would not do anything at the moment, but I would watch the skin colour very closely
5. I would consult the midwife
6. I would consult the midwife for bilirubin quantification
7. I would consult the midwife immediately for urgent bilirubin quantification
8. Other, please specify:
9. If bilirubin was quantified in this baby, what level of bilirubin in the blood would you expect?
10. Less than 50 µmol/L (<2.92 mg/dL)
11. Between 50 and 100 µmol/L (2.92 – 5.85 mg/dL)
12. Between 100 and 200 µmol/L (5.85 – 11.7 md/dL)
13. Between 200 and 300 µmol/L (11.7 – 17.54 mg/dL)
14. Between 300 and 450 µmol/L (17.54 – 26.32 mg/dL)
15. More than 450 µmol/L (>26.32 mg/dL)
