# Supplementary material for: Assessing knowledge and skills of maternity care professionals regarding neonatal hyperbilirubinaemia: a nationwide survey
Source: BMC Pregnancy Childbirth. 2021 Jan 19;21:63. doi: 10.1186/s12884-020-03463-0 (PMC7814718; doi:10.1186/s12884-020-03463-0)
Supplement: Supplementary file 3 — Additional file 3: Supplementary table 1. Frequencies of age and province for maternity care assistants in the national survey sample and in the Netherlands. [file 12884_2020_3463_MOESM3_ESM.docx]

**Additional file 3**

**Supplementary table 1: Frequencies of age and province for maternity care assistants in the national survey sample and in the Netherlands**

|  | MCA respondents in national survey sample (%) | All MCAs in the Netherlands (%) |
| --- | --- | --- |
| Age in years |  |  |
| <20 | 0 | 0 |
| 20-24 | 4 | 6 |
| 25-29 | 4 | 8 |
| 30-34 | 5 | 8 |
| 35-39 | 8 | 11 |
| 40-44 | 10 | 10 |
| 45-49 | 15 | 15 |
| 50-54 | 19 | 16 |
| 55-59 | 22 | 17 |
| 60-64 | 11 | 8 |
| ≥65 | 2 | 1 |
| Province of working area |  |  |
| Zuid-Holland | 23 | 21 |
| Noord-Holland | 15 | 16 |
| Noord-Brabant | 15 | 15 |
| Gelderland | 11 | 12 |
| Overijssel | 7 | 7 |
| Utrecht | 7 | 8 |
| Limburg | 6 | 7 |
| Friesland | 5 | 4 |
| Flevoland | 3 | 2 |
| Groningen | 2 | 3 |
| Drenthe | 3 | 3 |
| Zeeland | 2 | 2 |

MCA = maternity care assistant
