# Supplementary material for: Assessing knowledge and skills of maternity care professionals regarding neonatal hyperbilirubinaemia: a nationwide survey
Source: BMC Pregnancy Childbirth. 2021 Jan 19;21:63. doi: 10.1186/s12884-020-03463-0 (PMC7814718; doi:10.1186/s12884-020-03463-0)
Supplement: Supplementary file 4 — Additional file 4: Supplementary table 2. Comparison of question sets of training session questionnaire. [file 12884_2020_3463_MOESM4_ESM.docx]

**Additional file 4**

**Supplementary table 2: Comparison of question sets of training session questionnaire**

|  | Set A in part I  N = 53 | Set B in part I  N = 46 |
| --- | --- | --- |
| Pre-training score; median (IQR) | 5 (1) | 5 (2) |
| Age in years; mean (SD) | 45 (10) | 44 (11) |
| Experience as MCA in years; mean (SD) | 17 (9) | 18 (11) |
|  | *n (%)* | *n (%)* |
| Participated in e-learning before training session |  |  |
| Yes, completed | 40 (76) | 31 (67) |
| Yes, only partially completed | 6 (11) | 6 (13) |
| No | 7 (13) | 9 (20) |
| Location of training |  |  |
| Birth Hotel Maasstad | 19 (36) | 18 (39) |
| Birth Hotel Haga | 13 (25) | 11 (24) |
| Maternity Care Hotel Noord | 9 (17) | 8 (17) |
| Birth Clinic Westeinde | 8 (15) | 6 (13) |
| Birth Centre Sophia | 4 (8) | 3 (7) |

MCA = maternity care assistant; IQR = interquartile range; SD = standard deviation.
